# Negative correlation between central sensitization and forgotten joint score-12 after total hip arthroplasty

**DOI:** 10.1186/s13018-023-04175-9

**Published:** 2023-09-15

**Authors:** Takashi Imagama, Tomoya Okazaki, Yuta Matsuki, Takehiro Kaneoka, Takehiro Kawakami, Kazuhiro Yamazaki, Takashi Sakai

**Affiliations:** Department of Orthopaedic Surgery, Yamaguchi University Graduate School of Medicine, 1-1-1, Minamikogushi, Ube, 7558505 Japan

**Keywords:** Central sensitization, Forgotten joint score-12, Pain, Total hip arthroplasty

## Abstract

**Background:**

Central sensitization is a condition in which even mild stimuli cause pain due to increased neuronal reactivity in the dorsal horn of the spinal cord. It is one of factors of chronic pain in patients with osteoarthritis. However, it is unknown whether central sensitization relates to clinical outcomes after total hip arthroplasty (THA). This study aimed to clarify whether preoperative central sensitization relates to the forgotten joint score-12 (FJS) after THA. Moreover, the secondary outcome was to identify which items in the FJS were most related by central sensitization.

**Methods:**

This retrospective analysis included 263 hips (263 patients; 51 males and 212 females) that underwent primary THA in our institute and were available for evaluation one year postoperatively. The average patient age was 64.8 ± 11.9 years. The Central Sensitization Inventory (CSI) part A, which is a patient-reported outcome, was used to measure preoperative central sensitization. The correlation between preoperative CSI and postoperative FJS and the association between postoperative FJS and preoperative CSI severity were determined. Moreover, difference in each FJS item was evaluated by CSI severity.

**Results:**

Twenty-six patients (9.9%) had central sensitization preoperatively. Preoperative CSI scores and postoperative FJS were negatively correlated (*r* =  − 0.331, *p* < 0.0001). The postoperative FJS was significantly lower in patients with moderate or higher preoperative CSI severity than that in patients with subclinical or mild preoperative CSI severity (*p* < 0.05). FJS items with movement of daily life were significantly worse in the moderate or higher CSI severity compared with subclinical group (*p* < 0.05 or *p* < 0.01).

**Conclusion:**

Central sensitization prior to THA negatively related to postoperative FJS. In particular, the relationship of central sensitization was found to be significant in FJS items with movement, which would lead to lower patient satisfaction after THA. To get better postoperative outcomes in patients with preoperative central sensitization, improving central sensitization would be important.

## Background

Total hip arthroplasty (THA) for hip disease results in good long-term postoperative outcomes and has been described as the most successful surgery of the twentieth century [1]. However, a systematic review reported that 7–23% of patients had residual moderate or worse pain after THA [2], which is a major problem related to postoperative patient satisfaction.

In patients with osteoarthritis nociceptive pain due to mechanical stimulation or inflammation in joints is main symptom, which excites nociceptors in peripheral nerve fiber endings. The pain signals are subsequently transmitted from peripheral nerves to the cerebrum via the spinal cord. Thus, mechanical irritation and inflammation are reduced after THA, resulting in an improvement in pain. On the other hand, central sensitization is caused by an increase in the responsiveness of nociceptive neurons to normal or subthreshold afferent inputs in the central nervous system [3], and it has been attracting the attention of researchers in recent years. One proposed mechanism of the central sensitization is repeated stimulation due to chronic pain, which leads to structural, chemical and functional changes in the central nervous system. Finally, a state of increased neuronal reactivity was maintained without sensory stimulation. [4, 5]. Zolio et al. reported that 24–48% of patients with knee osteoarthritis develop central sensitization [6]. Although central sensitization is expected to be related to postoperative residual pain after THA, the frequency of preoperative central sensitization in patients undergoing THA and the involvement of central sensitization in postoperative outcomes have not been sufficiently clarified.

It was hypothesized that patients with central sensitization before THA would have lower postoperative FJS. In this study, the presence or absence of preoperative central sensitization was evaluated, and the aim was to clarify whether preoperative central sensitization relates to postoperative FJS in patients with THA. Moreover, specific items of the FJS that were related by preoperative central sensitization were also identified. The knowledge of these results would be useful in formulating treatment strategies to improve postoperative outcomes after THA.

## Methods

### Patients and study design

A total of 321 hips (308 patients) that underwent primary THA at our hospital from October 2018 to March 2022 were retrospectively enrolled in this study. Patients with terminal stage osteoarthritis of the hip on the non-operative side (14 hips), simultaneous bilateral primary THA (26 hips), postoperative dislocation (1 hip), and postoperative deep surgical site infection (1 hip) were excluded from the study. Moreover, the exclusion criteria included hip pain visual analogue scale (VAS) score of 20 mm or more on the non-operative side (8 hips) because it was reported that pain on the non-operative hip influenced FJS after THA [7]. Patients administered duloxetine preoperatively (4 hips) and those for whom PROMs could not be evaluated (4 hips) were also excluded, because it was reported to duloxetine could improve pain of central sensitization [8]. Finally, a total of 263 hips (263 patients; 51 males and 212 females) were included in the study. Age, sex, body mass index (BMI), and primary disease were extracted from the electronic medical records as demographics of these patients. Mean patient age and BMI were 64.8 ± 11.9 years and 24.7 ± 4.2 kg/m^2^ respectively. The primary diseases were osteoarthritis of the hip (225 hips), osteonecrosis of the femoral head (26 hips), rapidly destructive coxopathy (7 hips), and subchondral insufficiency fracture (5 hips). The posterior approach was used in 148 hips, the direct anterior approach in 103 hips, and the modified Watson-Jones approach in 12 hips. The statistical sample power using G*power 3.1.9.7 (Düsseldorf, Germany) was 0.96.

The study was approved by the Ethics Committee and Institutional Review Board at Yamaguchi University (H2020-068). We informed all patients about the study and obtained their consent.

### Evaluation criteria and methods

Several methods for assessing central sensitization have been reported. Among them, the Central Sensitization Inventory (CSI) is widely used because it is self-administered and easy to implement [9]. The CSI part A was used to evaluate preoperative central sensitization just before the surgery. The CSI part A includes 25 questions with a minimum score of 0 and a maximum score of 100. The severity levels of CSI are defined as subclinical (0–29 points), mild (30–39 points), moderate (40–49 points), severe (50–59 points), and extreme (60–100 points). Patients scoring 40 points or more (moderate or higher) are considered to have central sensitization [10, 11]. Currently, patient-reported outcome measures (PROMs) are becoming more important in the evaluation of THA outcomes [12]. Recently, the usefulness of the forgotten joint score-12 (FJS), a PROM based on the ultimate goal of making the patient unaware of the artificial joint in daily life, has been reported [13]. The score consists of 12 items regarding whether or not the patient is aware of their artificial joints in various situations in their daily lives. While it is difficult for other PROMs such as Oxford hip score to evaluate the small difference between good and excellent scores due to ceiling effect, the FJS is considered useful for such an evaluation [13]. Thus, we believe that it makes the most sense to evaluate outcome after THA with the FJS. The FJS was used to evaluate patients during an outpatient visit one year after THA. The CSI and FJS were filled out by the patients themselves. The correlation between one-year postoperative FJS and each of age, BMI, and preoperative CSI scores was investigated retrospectively. Age, sex, BMI, primary disease, surgical approach, and postoperative FJS were compared based on preoperative CSI severity (subclinical, mild, and moderate or higher groups). Each FJS item was compared between subclinical and moderate or higher groups of CSI severity. For each FJS item, never is 0 point, almost never is 1 point, seldom is 2 points, sometimes is 3 points, and mostly is 4 points, and 0 point is the best and 4 points is the worst score.

### Statistical analysis

Spearman’s rank correlation coefficient was used to determine the correlation between postoperative FJS and each of age, BMI, and preoperative CSI scores. The Kruskal–Wallis test was used to compare age, sex, BMI, primary disease, surgical approach, and postoperative FJS between the three groups according to preoperative CSI severity. The Mann–Whitney U test was used to analyze the scores of each FJS item between preoperative CSI severity. Continuous variables are expressed as mean and standard deviation, and categorical variables are expressed as number and percentage. Statistical significance was set at *p* < 0.05. The statistical analyses were conducted using GraphPad Prism version 8 (San Diego, CA, USA).

## Results

**Fig. 1:**
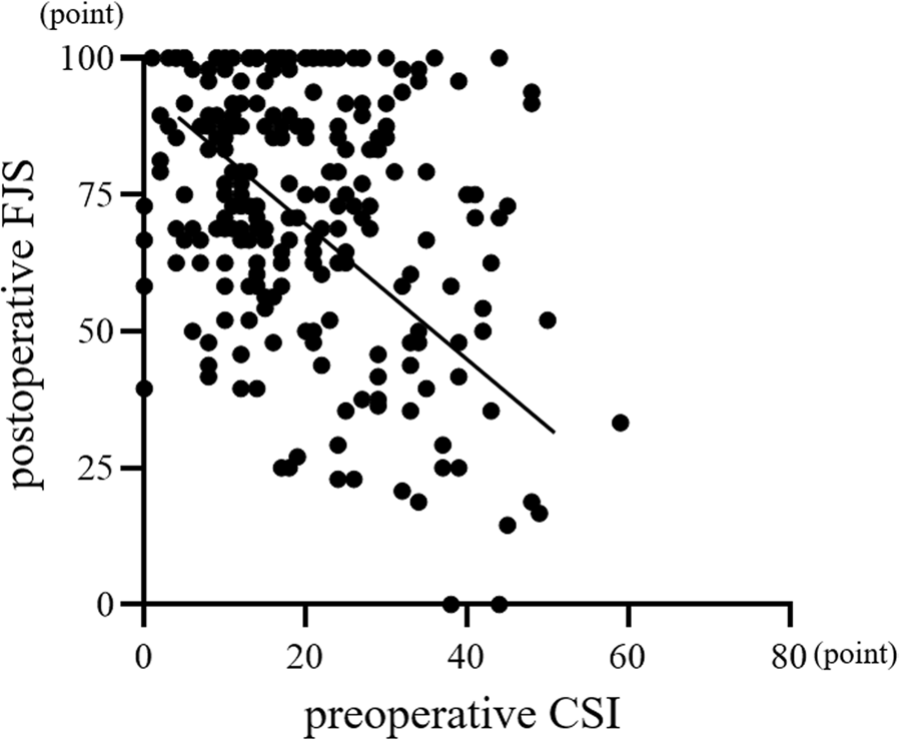
Preoperative CSI scores and postoperative FJS were negatively correlated (*r* = -0.331, *p* < 0.0001)

**Fig. 2:**
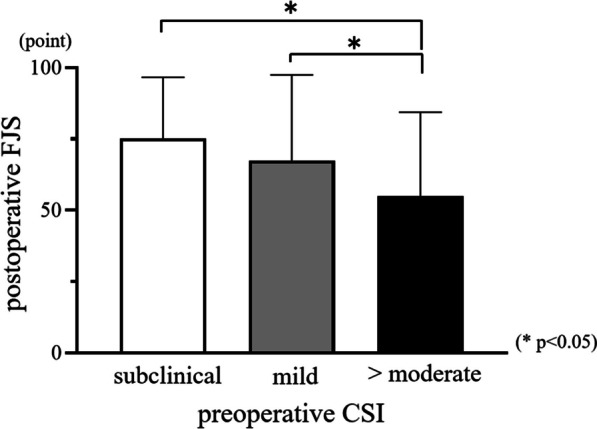
The postoperative FJS was significantly lower in patients with moderate or higher severe preoperative CSI score than that in patients with subclinical group or mild preoperative CSI scores (*p < 0.05)

**Fig. 3:**
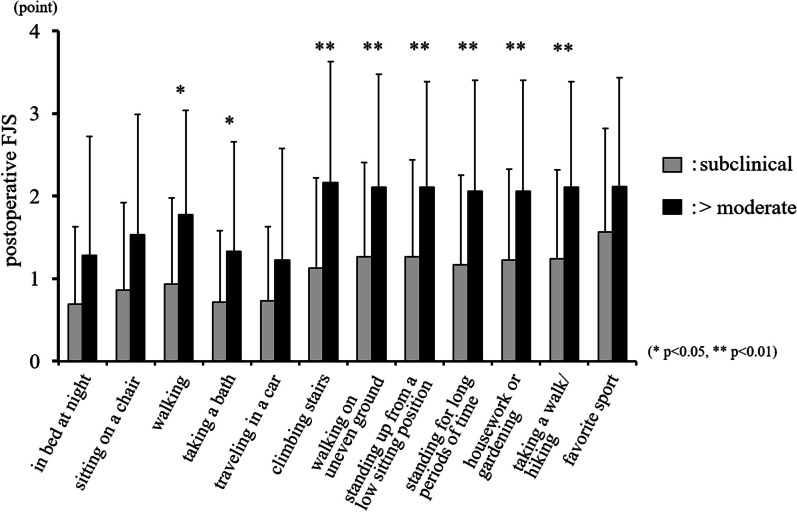
Each FJS item was compared between subclinical and moderate or higher CSI severity. Patients with moderate or higher CSI severity had significantly worse score mainly for the items with movement of daily life. (*p < 0.05, **p < 0.01)

The mean preoperative CSI score was 19.8 ± 11.3 points, and the mean postoperative FJS was 73.1 ± 22.9 points. Preoperative CSI was subclinical in 204 patients (77.6%), mild in 33 patients (12.5%), moderate in 24 patients (9.1%), and severe in 2 patients (0.8%). No patients were classified as having extreme central sensitization preoperatively. Conclusively, 26 patients (9.9%) had central sensitization. The preoperative CSI score was negatively correlated with postoperative FJS (*r* =  − 0.331, *p* < 0.0001) (Fig. 1). Age and BMI did not correlate with postoperative FJS (*r* =  − 0.125, *p* = 0.074, *r* =  − 0.002, *p* = 0.979, respectively). Patient age, sex, BMI, primary disease, and surgical approach were not significantly different between groups of patients with subclinical, mild, and moderate or higher CSI scores preoperatively (*p* = 0.913, 0.875, 0.424, 0.095, and 0.485 respectively) (Table 1). The postoperative FJS was significantly lower among patients with at moderate or higher preoperative CSI scores than among patients with subclinical or mild preoperative CSI scores (*p* < 0.05) (subclinical: 75.2 ± 21.4, mild: 67.4 ± 30.1, and > moderate: 54.9 ± 29.5). The FJS of the subclinical and mild CSI score groups were not significantly different (Fig. 2). Patients with moderate or higher CSI score preoperatively had significantly lower scores than those with subclinical preoperative CSI score for the following FJS items: walking, taking a bath, climbing stairs, walking on uneven ground, standing up from low-sitting position, standing for long periods of time, housework or gardening, and hiking (walking and taking a bath: *p* < 0.05; all others: *p* < 0.01) (Fig. 3).

**Table 1:** Patient demographics classified by CSI severity

CSI severity	Subclinical	Mild	> Moderate	*p* Value
Number (%)	204 (77.6)	33 (12.5)	26 (9.9)	N/A
Age (year)	64.8 ± 12.5	65.1 ± 10.4	64.5 ± 12.8	0.913
Sex (male: female)	39:165	6:27	6:20	0.875
BMI (kg/m^2^)	24.5 ± 4.2	23.8 ± 3.3	25.8 ± 5.1	0.424
Diagnosis (hips) OA:ONFH:RDC:SIF	176:22:5:1	28:2:1:2	21:2:1:2	0.095
Surgical approach (hips) PA:DAA:mWJA	110:84:10	20:11:2	18:8:0	0.485

*CSI* Central sensitization inventory, *BMI* body mass index, *OA* Osteoarthritis of the hip, *ONFH* Osteoarthritis of the femoral head, *RDC* rapidly destructive coxopathy, *SIF* subchondral insufficiency fracture, *PA* posterior approach, *DAA* direct anterior approach, *mWJA* modified Watson-Jones approach, *N/A* not applicable

## Discussion

Preoperative CSI scores and postoperative FJS were negatively correlated in patients underwent THA, and the FJS was the lowest in patients with moderate or higher preoperative CSI severity. That is to say, this study found that the presence of preoperative central sensitization would have negative correlation on postoperative outcome in patients with THA. Particularly, the items of FJS with movement had poor outcome due to central sensitization. This is the first study to report the correlation between CSI and FJS in patients with THA.

Izumi et al. reported that preoperative central sensitization by temporal summation in patients undergoing THA was correlated with the hip pain VAS scores at six weeks postoperatively and that central sensitization played a role in residual pain after THA [14]. In present study, central sensitization was observed in 9.9% of patients prior to THA, according to the CSI scores. And the presence of preoperative central sensitization related to the postoperative FJS. These indicated that central sensitization played a significant role in the pre- and postoperative symptoms of patients with THA. Moreover, the evaluations for FJS items regarding movement were significantly worse in patients with preoperative central sensitization. In contrast, a previous study reported that central sensitization affects pain VAS scores at rest more than pain VAS scores during activity in patients prior to THA [15]. The most likely reason for this discrepancy may be whether the evaluation is before or after THA. The influence of central sensitization may be masked preoperatively as the stimuli from nociceptive stimuli receptors in the deformed joint were too strong during activity. On the other hand, small stimuli during activity after THA would be amplified in patients with central sensitization because the nociceptive stimulus are considered negligibly small. It may have caused residual pain and affect the items of FJS with movement.

In this study, it was found that preoperative central sensitization had negative effect on the FJS after THA. Reducing pain due to central sensitization preoperatively may improve the postoperative FJS. Koh et al. randomly divided patients with central sensitization before total knee arthroplasty (TKA) into an oral duloxetine group and a control group and found that postoperative pain VAS scores were significantly lower among patients who were administered duloxetine [8]. This effect would be due to activation of the descending pain inhibition system. Serotonin and noradrenaline are involved in pain inhibition through the activation of the descending pain inhibitory system in the brain and spinal cord, and duloxetine activates the descending pain inhibitory system via serotonin and noradrenaline re-uptake inhibition [16]. This mechanism may reduce postoperative pain in patients with central sensitization who undergo TKA. Although no studies regarding the effects of duloxetine on postoperative pain or FJS in patients undergoing THA have been reported, duloxetine is expected to be sufficiently effective to reduce pain in this patient population. Although further research is required in the future, such treatment for central sensitization before and after surgery may lead to improved outcomes after THA. Other treatments for central sensitization include educating patients, exercise, and cognitive behavioral therapy. Patients with central sensitization often have inappropriate perceptions of pain, believing that something terrible is happening in their body, leading to poor function and lower quality of life (QOL) [17, 18]. A randomized controlled trial showed that education about the pain physiology corrected the perception of pain, and it leads to deceased pain, better physical function and mood, and increased energy [19]. Exercise has also been shown to be beneficial for chronic pain patients. Ambrose and Golightly reported that exercise not only improves pain, but also physical function, sleep, and cognitive function [20]. However, when patients get into a cycle where excessive exercise worsens symptoms, they develop a condition called pain memory, which associates pain with exercise [21]. Thus, Nijs et al. recommend graded exercise based on goals, such as duration of exercise, number of repetitions, and distance. This can conduct new memory pathways in the brain and reduce the perception of pain and fear of movement [21]. Cognitive behavioral therapy is a technique to reduce pain by reframe negative thoughts, emotions, and behaviors as positive ones. Acceptance and commitment therapy focuses less on controlling negative thoughts and behaviors and more on helping patients accept and overcome negative obstacles such as pain and central sensitization. Significant results have been reported with this approach, including improved overall QOL for patients [22, 23]. These approaches may also be useful for pre- and postoperative THA patients with central sensitization.

This study has several limitations. First, the number of patients with moderate or higher preoperative central sensitization is small. However, a post-hoc test conducted in the subclinical group and the moderate or higher group using G*power 3.1.9.7 (Düsseldorf, Germany) revealed a sample power of 0.96, classifying the current study as a valuable evaluation. Second, although there were no significant differences in age, sex, BMI, surgical approach, and primary disease between the groups, some papers reported that surgical approaches affect postoperative FJS after THA [24, 25]. On the contrary, other reports showed no difference in postoperative FJS between surgical approaches [26, 27]. Thus, we consider that whether the surgical approach affects FJS is controversial. It is also possible that the duration of pain before the surgery might differ from disease to disease, which might affect central sensitization. Arendt-Nielsen et al. reported an association between a longer symptom and central sensitization [28]. On the other hand, Neogi et al. described that there was no relationship between the duration of illness and central sensitization [29]. That is to say, it is controversial at present. Therefore, multiple primary diseases were allowed to be included in this study. Other demographics of patients might have differed slightly between the groups. Particularly, other joint pain or low back pain affected postoperative FJS in patients with THA [7, 30]. Thus, in present study, the patients with painful non-operative hip were excluded. However, the evaluation of low back pain was not conducted. Further study will be needed. Finally, the follow-up observation period was relatively short. In this study, because the evaluations were conducted up to one year postoperatively, the long-term effects of central sensitization were not revealed. A large-scale randomized controlled study with a long-term postoperative evaluation is necessary.

## Conclusion

This study investigated the relationship between preoperative CSI and postoperative FJS at one year after THA, as preoperative central sensitization may be a cause of residual pain after THA. Preoperative CSI score was negatively correlated with postoperative FJS, and patients with moderate or higher preoperative CSI severity had significantly lower FJS. Particularly, the scores of FJS items with movement of daily life were worse in these patients. These results suggest that preoperative central sensitization relates to the clinical outcomes after THA. Therefore, it would be important for better clinical outcomes after THA to improve preoperative central sensitization.

## Data Availability

The datasets used and/or analyzed during the current study are available from the corresponding author on reasonable request.

## Abbreviations

THA: Total hip arthroplasty
PROMs: Patient-reported outcome measures
FJS: Forgotten joint score-12
CSI: Central sensitization inventory
VAS: Visual analogue scale
BMI: Body mass index
TKA: Total knee arthroplasty
